# Correlation Between Technology and Improved Outcomes in Youth With Type 1 Diabetes Mellitus: Prospective Study Examining Outcomes for Patients With Depression and Those With Public Insurance

**DOI:** 10.2196/70380

**Published:** 2025-06-03

**Authors:** Natacha D Emerson, Christopher Ferber, Nicholas J Jackson, Joshua Li, Eric Tsay, Dennis Styne, Michael Gottschalk, Steven D Mittelman, Anna-Barbara Moscicki

**Affiliations:** 1 Department of Psychiatry and Biobehavioral Sciences, David Geffen School of Medicine, University of California, Los Angeles, 760 Westwood Plaza, Ste 48-241, Los Angeles, CA, 90095, United States, 1 3107948416; 2 Department of Medicine, Data Management and Statistical Core, David Geffen School of Medicine, University of California, Los Angeles, Los Angeles, CA, United States; 3 Division of General Internal Medicine and Health Services Research, Department of Medicine, University of California, Los Angeles, , Los Angeles, CA, United States, United States; 4 Department of Pediatrics, Division of Pediatric Endocrinology, Loma Linda University School of Medicine, Loma Linda, CA, United States; 5 University of California, Davis, Davis, CA, United States; 6 University of California, San Diego, San Diego, CA, United States

**Keywords:** adolescence, depression, health insurance, continuous glucose monitor, pump, adherence, type 1 diabetes mellitus, T1DM

## Abstract

**Background:**

Adherence to type 1 diabetes mellitus (T1DM) treatment regimens decreases during adolescence. While comorbid depression and health insurance disparities are individually known to potentiate this risk, technological devices for T1DM appear to be protective.

**Objective:**

We examined whether technology use impacted the association between depression and poorer health outcomes in T1DM. Given established insurance-based disparities based on technology access, we also studied whether the protective effects of T1DM technology differed among publicly and privately insured youth.

**Methods:**

Data were prospectively collected from pediatric patients with T1DM across 3 California medical centers. We used linear and negative binomial regression analyses to examine whether technology use was related to diabetes outcomes and whether this differed based on depression status (technology-by-depression interaction) and health insurance type (technology-by-insurance interaction).

**Results:**

Across 1573 patients aged 12 to 25 years (mean age 15.9, SD 2.9 years; n=1050, 66.4%, non-Hispanic White; n=745, 47.0% female), those with a depression diagnosis had higher hemoglobin A_1c_ (HbA_1c_; mean 9.1%, SD 2.1% vs 10.1%, SD 2.2%) and more frequent diabetic ketoacidosis (DKA) events per year (mean 0.10, SD 0.36 vs 0.24, SD 0.66) than those without (*P=.*003). Patients using both a continuous glucose monitor (CGM) and pump had lower HbA_1c_ levels and fewer DKA events per year (mean HbA_1c_ 8.2%, SE 0.1%; mean DKA events per year 0.05, SE 0.01) than those using one device (mean HbA_1c_ 9.0%, SE 0.1%; mean DKA events 0.08, SE 0.1%) or none (mean HbA_1c_ 10.0%, SE 0.1%; mean DKA events 0.19, SE 0.1%; *P*<.001). While youth with public insurance had significantly higher HbA_1c_ levels than those with commercial insurance (mean 9.3%, SD 2.1% vs 9.0%, SD 2.0%, *P*<.001), those using a CGM had no reliable decrease in HbA_1c_ compared to their commercially insured peers (*P*=.35).

**Conclusions:**

Technology use in pediatric T1DM appears protective for both youth with a history of depression and those who are publicly insured. These data underscore the importance of universal access to technology to mitigate disparities based on comorbid mental health issues and differential access to care.

## Introduction

The incidence of pediatric type 1 diabetes mellitus (T1DM) is increasing wordwide 3% per year [1]. In the United States, the cost of diagnosed diabetes was estimated to be in the US $400 billion range in 2022 [2], much of this cost being related to complications of suboptimal adherence to treatment. Consistent glucose control is vital to avoiding long-term complications of T1DM, such as retinopathy, nephropathy, and cardiovascular disease [3,4]. Current guidelines suggest keeping hemoglobin A_1c_ (HbA_1c_), the traditional gold standard metric for T1DM control, to below 7% in both children [5] and adults [6]. Another important clinical outcome in diabetes care is the prevention of diabetic ketoacidosis (DKA), a life-threatening complication of diabetes that continues to be the most common cause of hospitalization and death in children with T1DM [7,8].

The advent of insulin pumps and continuous glucose monitors (CGMs) has significantly advanced T1DM management in recent years. Children who use CGMs [9] and pumps [10] are more likely to attain HbA_1c_ levels below 7%, irrespective of socioeconomic status, insulin regimen, or duration of diabetes [9]. Despite these advancements, adherence to T1DM regimens (eg, consistent insulin administration, regular glucose monitoring, and appropriate compliance with dietary recommendations) continues to be suboptimal. The transition from childhood to adolescence has been established as a strong risk factor for poorer adherence and blood glucose control [11,12]. This developmental transition is characterized by shifting responsibilities from parents to patient, feelings of increased social pressure from peers, and fatigue from chronic illness management [13], factors that have been shown to further complicate this period and to be linked to comorbid depression. Depression is common in adolescence and has been linked to reduced adherence [14] and higher HbA_1c_ levels [15] via its negative impact on motivation, cognitive functioning, and self-efficacy.

Health insurance type is known to influence access to T1DM care and supplies [16]. Despite growing evidence that generous insurance coverage of diabetes technology improves outcomes [17], there continue to be significant barriers for youth with public insurance related to strict prior authorization requirements, high copays, and lack of access to specialized health care for both pumps and CGMs [16].

The aim of this study was to investigate whether diabetes technology use moderated the negative effects of teenage depression across 3 large health care systems in California. We also examined whether technology use mitigated the previously demonstrated disparities in glycemic control associated with public insurance. We hypothesized that having a history of depression and being publicly insured would predict higher HbA_1c_ levels and more frequent episodes of DKA, and that these effects would be reduced by the use of technology.

## Methods

### Participants

Data were prospectively collected from patients aged 12 to 25 years with T1DM who were seen for outpatient care at 1 of 3 University of California (UC)–affiliated health system pediatric endocrinology clinics between 2016 and 2021: UC Davis, UC Los Angeles, and UC San Diego.

### Ethical Considerations

The following work represents secondary analyses using existing clinical data with primary consent. All data were anonymized prior to analysis. The original consent for clinical care within the respective medical institutions covers secondary analyses without additional consent and therefore IRB approval was not required for this study.

### Data Collection

Health care providers were asked to complete flow sheets that were synchronized by the 3 pediatric endocrinology clinics to collect similar information at each clinical appointment. For patients with multiple time points, data associated with the most recent visit were used. The flow sheets contained demographic and medical data, such as age, sex, race/ethnicity, health insurance type (public [MediCal, California Children’s Services] versus commercial [health maintenance organization, preferred provider organization]), diabetes technology use (pump, CGM, neither, or both), number of DKA events in the past year, most recent HbA_1c_ level, and depression diagnosis. While some centers established a depression diagnosis based on a standard depression screener (eg, the Patient Health Questionnaire 9), other patients were asked to self-report an existing depression diagnosis.

### Statistical Analysis

Demographic differences between patients with and without a depression diagnosis were assessed using the Welch *t* test and a *χ*^2^ analysis. Multivariable associations of technology use and depression on HbA_1c_ levels and number of DKA events were assessed using linear and negative binomial regression models, respectively. Models were adjusted for age, sex, and insurance status (public vs private). To test for differential impacts of technology use on patients with and without depression, interactions terms for technology-by-depression were introduced into these models. Analyses were conducted in R (version 4.1.3; R Project for Statistical Computing) and Stata (version 17; StataCorp).

## Results

### Overview

There were 15,284 flow sheets for the patients, aggregated to a person-year level. Of these, 35.50% (n=5426) were missing information on depression, 6635 (43.41%) were missing information on HbA_1c_ level or DKA events, 112 (0.73%) were missing CGM or pump use information, and none were missing covariate information (age, sex, and insurance status). Our final analytic sample size was 2896 person-years from 1573 patients (average 1.8 years, SD 0.99 contributed per person). Of the contributed person-years, 2089 (72.1%) were from UC San Diego, 567 (19.6%) were from UC Davis, and 240 (8.3%) were from UC Los Angeles. The average age of all patients was 15.9 (SD 2.9) years, and patients were evenly split by sex and public versus private insurance (Table 1). There were no significant differences in health outcomes by age (mean difference 0.3, SD 3.0 years; *P*=.27) or sex (n=1426, 52.84% vs n=95, 48.22% for boys; *P*=.24). As has been previously reported, patients who identified as Hispanic and Black had higher HbA_1c_ and more frequent DKA events (Table 1).

**Table 1.** Demographic characteristics of study participants stratified by depression status. Significant *P* values are shown in italics.

Variables		Not depressed (n=2699, 93%)	Depressed (n=197, 7%)	*P* value
**Sex, n (%)**				.24
	Female	1269 (47.03)	102 (51.78)	
	Male	1426 (52.84)	95 (48.22)	
Age (years), mean (SD)		16.0 (2.9)	15.7 (3.0)	.27
**Insurance type, n (%)**				.51
	Private	1348 (49.96)	94 (47.72)	
	Public	1347 (49.92)	103 (52.28)	
Hemoglobin A_1C_ (%), mean (SD)		9.10 (2.05)	10.11 (2.22)	*<.001*
Diabetic ketoacidosis events per year per patient, mean (SD)		0.104 (0.364)	0.244 (0.658)	*.003*
**Race/ethnicity, n (%)**				*.02*
	Asian	90 (3.34)	2 (1.02)	
	Black non-Hispanic	144 (5.34)	7 (3.55)	
	Hispanic	806 (29.88)	80 (40.61)	
	Other	284 (10.52)	22 (11.17)	
	White non-Hispanic	1320 (48.91)	82 (41.62)	
	Unknown	51 (1.89)	4 (2.03)	
	CGM^a^ use (yes)	1270 (47.07)	75 (38.07)	*.01*
	Pump use (yes)	1125 (41.70)	73 (37.06)	.19
**Technology use, n (%)**				.06
	No technology	1033 (38.26)	89 (45.18)	
	1 technology (CGM or pump)	929 (34.45)	68 (34.52)	
	2 technologies (CGM and pump)	733 (27.16)	40 (20.30)	

a CGM: continuous glucose monitor.

### Technology Use

Use of either CGM (dichotomized as yes/no; mean HbA_1c_ level 8.5%, SD 1.7% vs 9.8%, SD 2.2%) or pump (dichotomized as yes/no; mean HbA_1c_ level 8.6%, SD 1.6% vs 9.6%, SD 2.3%) technology was associated with lower HbA_1c_ levels (*P*<.001). When comparing use of 0, 1, or 2 forms of technology, use of *both* CGM and a pump was associated with more significant reductions in HbA_1c_ than either technology alone (mean HbA_1c_ level 8.2%, SD 1.4% vs. 9.1%, SD 2.1%; *P*<.001). A similar pattern was found for DKA events (mean DKA events 0.07, SD 0.25 vs 0.15, SD 0.48 for CGM technology; mean DKA events 0.05, SD 0.27 vs 0.15, SD 0.45 for pump technology, mean DKA events 0.05, SD 0.23 vs 0.08, SD 0.31 for one compared to both technologies; *P*<.001).

### Technology Use and Depression

Patients with depression had significantly higher HbA_1c_ and more DKA events relative to nondepressed patients (Table 1). There was a main effect of technology use across diabetic outcomes for both patients with depression and those without, such that youth with depression who used a CGM, pump, or both had lower HbA_1c_ and fewer DKA events than youth with depression who used no technology (*P*<.01 for all comparisons; Multimedia Appendix 1)*.* The same pattern was found in patients without a history of depression (*P*<.001 for all comparisons; Multimedia Appendix 1).

### Technology Use and Insurance Type

**Figure 1.**
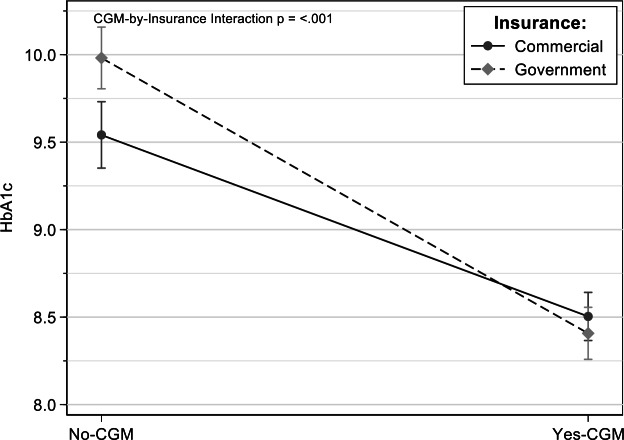
Hemoglobin A_1C_ (HbA_1C_) levels for youth with and without use of a continuous glucose monitor along insurance types.

There were significant differences in diabetic outcomes based on insurance type. Youth with public insurance had higher HbA_1c_ levels than those with commercial health insurance (*P*<.001). Youth with public insurance who used a CGM had similar mean HbA_1c_ levels as youth with commercial insurance who used a CGM, effectively reversing the disparity in health outcomes associated with public insurance (interaction: *P*<.001; Figure 1). Using a pump was also associated with significantly lower HbA_1c_ levels for both commercially and publicly insured youth, though the pump-by-insurance type interaction was not significant *(P*=.30). The relationship between technology use and insurance type was not significant for DKA events *(P*<.05; Multimedia Appendix 2).

## Discussion

We found that use of diabetes technology was associated with improved glycemic control, attenuating the negative impact of depression and mitigating outcome disparities related to public insurance. Diabetes technology use was predictive of better glycemic control through both lower HbA_1c_ levels and fewer DKA events. While having a reported depression diagnosis was associated with higher HbA_1c_ levels and more DKA events, technology use attenuated this risk in an additive manner. These findings support the strategy of offering diabetes technology to all youth, regardless of comorbid mental health issues. However, providing an insulin pump to youth with depression has historically been considered risky given the potential for self-harm via exogenous insulin administration. Given the benefits of technology use in youth with depression, clinicians who continue to be hesitant to prescribe a pump to due to fears of “suicide by insulin*”* should consider using tailored screening of suicide risk rather than a broader depression or mood measure. The Columbia-Suicide Severity Rating Scale [18] appears to identify twice the number of T1DM patients at risk for self-harm than its more general depression counterpart, the Patient Health Questionnaire 9 [19], which was used in this study [20]. Our results also underline the importance of screening for depression in pediatric settings in order to minimize its impact on the health of individuals with T1DM. In fact, research shows that both pharmacological and psychotherapeutic treatments for depression are associated with improvements in glycemic control [21].

Although not examined in this study, it is also worthwhile to consider whether comorbid depression impacts regularity and consistency of technology use. In general, youth who perceive more barriers to technology use are less likely to use it [22]. In youth with T1DM, commonly reported barriers to consistent technology use include cost and insurance issues and wear-related concerns [22]. While depression does not appear to predict discontinuation of CGM use [23], more research is needed to confirm this finding. Data on technology use collected for this study reflect a dichotomous variable and therefore cannot be used to inform the larger literature on health outcomes as it relates to technology consistency and discontinuation. What is known in regards to CGM is that users consistently report more engagement in diabetes self-care and a better quality of life, likely because they feel more efficacious in day-to-day disease management [24]. Given that helplessness is central to the development and maintenance of depression [25], encouraging CGM use may present one way to help youth feel more in control of their health.

With regard to insurance type, our study demonstrates that outcome disparities between publicly and commercially insured youth are mitigated by both CGM and pump use. Prior studies of publicly insured pediatric patients with T1DM indicate that interruptions in the use of CGM were largely due to insurance gaps and were predictive of poorer outcomes [26]. Countries that have facilitated technology access have seen improved clinical outcomes in youth with T1DM. For instance, after Australia changed its national health policy to universally subsidize CGM use in youth with T1DM, both HbA_1c_ levels and DKA hospitalizations significantly decreased in youth [27]. There have also been documented differences in countries with and without universal health care. A large-scale examination of T1DM outcomes in American versus German youth found that disparities between socioeconomic status and HbA_1c_ were evident in both groups but that technology access was a significant covariate only in the American sample [28].

At the time of data collection in California (2016-2021), public insurance companies mandated that patients document 4 fingerstick blood glucose tests every day for at least 1 month to qualify for a CGM [16]. Even 1 instance of a missed test could disqualify families. Many other state Medicaid programs have had the same requirement [16]. Fortunately, as of 2021, pharmacy benefits for publicly insured patients in California have expanded, making it easier to obtain diabetes technology for publicly insured patients [29]. Our data support expanding pharmacy benefits to other states that have not yet made them publicly available.

Besides improved clinical outcomes, prior research has shown that facilitating universal access to CGM and pump use is associated with significant cost savings. While the cost of a CGM is estimated to be US $15.20/day (extrapolated to approximately US $5000/year) [30], 1 admission for DKA in the United States costs upward of $30,000 [31]. In fact, the cost-effectiveness ratio of CGMs in pediatric T1DM is well established, demonstrating not only short-term improvements but significant reductions in costs related to long-term complications [32], emergency room visits, and hospitalizations [33].

There are several limitations in our current study. First and foremost, as this was a retrospective, nonrandomized cohort, causality cannot be inferred from our results. Patients who use diabetes technology were self-selected to some degree, and thus may have had differences in motivation, self-efficacy, and other important variables that could contribute to improved outcomes. Patients and families more motivated to attain optimal diabetes control may be more likely to pursue technology, suggesting that social support is also likely at play. Therefore, future studies evaluating social determinants of health may be helpful to clarify the impact of social support on the benefits of diabetes technology. As laid out in the introduction, publicly insured youth with CGM may have had to document daily adherence to blood glucose monitoring in order to qualify for the device. As such, we cannot exclude the possibility that HbA_1c_ levels for this group are attributable to better overall adherence rather than solely contingent on subsequent CGM use.

Depression was collected as a binary variable, and so degree of depression and status of treatment were not known. Patients with milder or better-controlled depression might be more likely to agree to diabetes technology than those with more severe symptomatology (due to increased feelings of hopelessnes and helplessness). Furthermore, the depression diagnosis data were not collected uniformly, which could have impacted the validity of results. It is also possible that some patients in the sample may have had undiagnosed depression, while others may have said they had depression without having a formal diagnosis. Future research should seek to replicate these results using a uniform method of depression screening. Thankfully, the utility of universal depression screening in pediatric patients with T1DM is gaining momentum [34]. It is also worth considering preliminary data on the use of medical record mining algorithms that can effectively predict patients at higher risk of depression in pediatric settings, where adolescents may be likely to underreport difficulties due to stigma, socially desirable responding, and the desire to be autonomous in their health management [35]. The integration of mental health care providers in clinical settings can also be a value-added service that removes the burden of mental health screening from endocrinologists [36].

To conclude, this is the first study to examine the interplay between socioeconomic, psychological, and medical factors in elucidating differential outcomes for adolescents with T1DM. It also represents a large sample of youth across 3 major health systems from one of the most populous states, allowing for the examination of these complex variables at the state level. Future studies should aim to replicate the above model with a national sample to determine how insurance status interacts with sociocultural variables such as parent education, income, race/ethnicity, and health literacy, especially given established disparities in technology acceptance based on cultural factors [37]. Our study also supports universal depression screening of adolescents in pediatric settings to ensure that mental health issues are treated promptly and are less likely to impact medical trajectories. Finally, given the concerning trend that socioeconomic status–based disparities appear to be increasing in American youth with T1DM [28], we cannot overstress the importance of facilitating universal coverage of diabetes technology. Such efforts are vital in our fight to provide equitable care that will facilitate improved clinical outcomes across demographic groups.

## Supplementary material

10.2196/70380 Multimedia Appendix 1 Adjusted effects of technology use on hemoglobin A_1c_ levels and diabetic ketoacidosis events for youth with and without depression.

10.2196/70380 Multimedia Appendix 2 Adjusted effects of insurance type on hemoglobin A_1c_ levels and diabetic ketoacidosis events by technology use.

## Abbreviations

CGM: continuous glucose monitor
DKA: diabetic ketoacidosis
HbA_1c_: hemoglobin A_1c_
T1DM: type 1 diabetes mellitus
UC: University of California

## References

[1] Kahkoska AR, Dabelea D (2021). Diabetes in youth: a global perspective. Endocrinol Metab Clin North Am.

[2] Parker ED, Lin J, Mahoney T (2024). Economic costs of diabetes in the U.S. in 2022. Diabetes Care.

[3] Rausch JR, Hood KK, Delamater A (2012). Changes in treatment adherence and glycemic control during the transition to adolescence in type 1 diabetes. Diabetes Care.

[4] Melendez-Ramirez LY, Richards RJ, Cefalu WT (2010). Complications of type 1 diabetes. Endocrinol Metab Clin North Am.

[5] American Diabetes Association Professional Practice Committee (2022). Children and adolescents: standards of medical care in diabetes-2022. Diabetes Care.

[6] Franz MJ, Bantle JM, Beebe CA (2002). American Diabetes Association position statement: evidence-based nutrition principles and recommendations for the treatment and prevention of diabetes and related complications. J Am Diet Assoc.

[7] Bialo SR, Agrawal S, Boney CM, Quintos JB (2015). Rare complications of pediatric diabetic ketoacidosis. World J Diabetes.

[8] Jensen ET, Stafford JM, Saydah S (2021). Increase in prevalence of diabetic ketoacidosis at diagnosis among youth with type 1 diabetes: the SEARCH for diabetes in youth study. Diabetes Care.

[9] Sanderson EE, Abraham MB, Smith GJ, Mountain JA, Jones TW, Davis EA (2021). Continuous glucose monitoring improves glycemic outcomes in children with type 1 diabetes: real-world data from a population-based clinic. Diabetes Care.

[10] Miller KM, Beck RW, Foster NC, Maahs DM (2020). HbA1c levels in type 1 diabetes from early childhood to older adults: a deeper dive into the influence of technology and socioeconomic status on HbA1c in the T1D Exchange Clinic Registry findings. Diabetes Technol Ther.

[11] Almeda-Valdes P, Palacio Ríofrio J, Zamudio Coronado KW (2019). Factors associated with insulin nonadherence in type 1 diabetes mellitus patients in Mexico. Dubai Diabetes Endocrinol J.

[12] Clements MA, Foster NC, Maahs DM (2016). Hemoglobin A1c (HbA1c) changes over time among adolescent and young adult participants in the T1D Exchange Clinic Registry. Pediatr Diabetes.

[13] Borus JS, Laffel L (2010). Adherence challenges in the management of type 1 diabetes in adolescents: prevention and intervention. Curr Opin Pediatr.

[14] Gonzalez JS, Peyrot M, McCarl LA (2008). Depression and diabetes treatment nonadherence: a meta-analysis. Diabetes Care.

[15] Bernstein CM, Stockwell MS, Gallagher MP, Rosenthal SL, Soren K (2013). Mental health issues in adolescents and young adults with type 1 diabetes: prevalence and impact on glycemic control. Clin Pediatr (Phila).

[16] Anderson JE, Gavin JR, Kruger DF (2020). Current eligibility requirements for CGM coverage are harmful, costly, and unjustified. Diabetes Technol Ther.

[17] Everett EM, Wisk LE (2022). Relationships between socioeconomic status, insurance coverage for diabetes technology and adverse health in patients with type 1 diabetes. J Diabetes Sci Technol.

[18] Posner K, Brent D, Lucas C (2008). Columbia-Suicide Severity Rating Scale (c-SSRS). Columbia University Medical Center.

[19] Kroenke K, Spitzer RL, Williams JB (2001). The PHQ-9: validity of a brief depression severity measure. J Gen Intern Med.

[20] Moss AC, Roberts AJ, Yi-Frazier JP (2022). Identifying suicide risk in adolescents and young adults with type 1 diabetes: are depression screeners sufficient?. Diabetes Care.

[21] van der Feltz-Cornelis C, Allen SF, Holt RIG, Roberts R, Nouwen A, Sartorius N (2021). Treatment for comorbid depressive disorder or subthreshold depression in diabetes mellitus: systematic review and meta-analysis. Brain Behav.

[22] Brew-Sam N, Chhabra M, Parkinson A (2021). Experiences of young people and their caregivers of using technology to manage type 1 diabetes mellitus: systematic literature review and narrative synthesis. JMIR Diabetes.

[23] Messer LH, Tanenbaum ML, Cook PF (2020). Cost, hassle, and on-body experience: barriers to diabetes device use in adolescents and potential intervention targets. Diabetes Technol Ther.

[24] Polonsky WH, Hessler D (2013). What are the quality of life-related benefits and losses associated with real-time continuous glucose monitoring? A survey of current users. Diabetes Technol Ther.

[25] Pryce CR, Azzinnari D, Spinelli S, Seifritz E, Tegethoff M, Meinlschmidt G (2011). Helplessness: a systematic translational review of theory and evidence for its relevance to understanding and treating depression. Pharmacol Ther.

[26] Addala A, Maahs DM, Scheinker D, Chertow S, Leverenz B, Prahalad P (2020). Uninterrupted continuous glucose monitoring access is associated with a decrease in HbA1c in youth with type 1 diabetes and public insurance. Pediatr Diabetes.

[27] Johnson SR, Holmes-Walker DJ, Chee M (2022). Universal subsidized continuous glucose monitoring funding for young people with type 1 diabetes: uptake and outcomes over 2 years, a population-based study. Diabetes Care.

[28] Addala A, Auzanneau M, Miller K (2021). A decade of disparities in diabetes technology use and HBa1c in pediatric type 1 diabetes: a transatlantic comparison. Diabetes Care.

[29] Yan K, Sainz N (2021). CGM and Medicaid: who’s covered?. DiaTribe change.

[30] Wan W, Skandari MR, Minc A (2018). Cost-effectiveness of continuous glucose monitoring for adults with type 1 diabetes compared with self-monitoring of blood glucose: the DIAMOND randomized trial. Diabetes Care.

[31] Desai D, Mehta D, Mathias P, Menon G, Schubart UK (2018). Health care utilization and burden of diabetic ketoacidosis in the U.S. over the past decade: a nationwide analysis. Diabetes Care.

[32] Huang ES, O’Grady M, Basu A (2010). The cost-effectiveness of continuous glucose monitoring in type 1 diabetes. Diabetes Care.

[33] O’Meara M, Mateus Acuña JC, Uribe A (2023). Long-term benefits of an integrated continuous glucose monitoring and insulin pump system for emergency admissions, hospitalization, and metabolic control in a cohort of people with diabetes: retrospective cohort study. JMIR Diabetes.

[34] Silverstein J, Cheng P, Ruedy KJ (2015). Depressive symptoms in youth with type 1 or type 2 diabetes: results of the Pediatric Diabetes Consortium Screening Assessment of Depression in Diabetes study. Diabetes Care.

[35] Jin H, Wu S (2019). Use of patient-reported data to match depression screening intervals with depression risk profiles in primary care patients with diabetes: development and validation of prediction models for major depression. JMIR Form Res.

[36] Heilbrun A, Drossos T (2020). Evidence for mental health contributions to medical care in diabetes management: economic and professional considerations. Curr Diab Rep.

[37] Loomba L, Bonanno S, Arellano D, Crossen S, Glaser N (2023). Disparities in insulin pump use among Spanish-speaking children with type 1 diabetes compared to their non-Hispanic White peers: mixed methods study. JMIR Diabetes.

