# Supplementary material for: Correlation Between Technology and Improved Outcomes in Youth With Type 1 Diabetes Mellitus: Prospective Study Examining Outcomes for Patients With Depression and Those With Public Insurance
Source: JMIR Diabetes. 2025 Jun 3;10:e70380. doi: 10.2196/70380 (PMC12151526; doi:10.2196/70380)
Supplement: Multimedia Appendix 1 [file diabetes-v10-e70380-s001.docx]

Table 2. Adjusted effects of technology use on HbA1c levels and DKA events for depressed and non-depressed youth

| **^a^HbA1c** | **Not Depressed** Est (95% CI) | **Depressed** Est (95% CI) | **Difference** Est (95% CI) | ***p*** |
| --- | --- | --- | --- | --- |
| No CGM | 9.7 (9.6, 9.8) | 10.6 (10.2, 11.1) | 0.9 (0.5, 1.4) | <.001 |
| CGM | 8.4 (8.3, 8.5) | 9.2 (8.7, 9.8) | 0.8 (0.3, 1.3) | 0.002 |
|  |  |  |  |  |
| *Difference p* | <.001 | <.001 | 0.74^1^ |  |
| No Pump | 9.5 (9.4, 9.6) | 10.7 (10.2, 11.1) | 1.2 (0.7, 1.6) | <.001 |
| Pump | 8.6 (8.4, 8.7) | 9.2 (8.7, 9.6) | 0.6 (0.2, 1.1) | 0.006 |
|  |  |  |  |  |
| *Difference p* | <.001 | <.001 | 0.10^1^ |  |
| No Technology | 9.9 (9.7, 10.0) | 10.9 (10.4, 11.5) | 1.1 (0.5, 1.6) | <.001 |
| 1 Tech | 8.9 (8.8, 9.1) | 9.9 (9.4, 10.4) | 1.0 (0.4, 1.5) | <.001 |
| 2 Tech | 8.2 (8.1, 8.3) | 8.6 (8.1, 9.2) | 0.4 (-0.1, 1.0) | 0.14 |
|  |  |  |  |  |
| *Difference (1 vs. None) p* | <.001 | 0.005 | 0.78^1^ |  |
| *Difference (2 vs. None) p* | <.001 | <.001 | 0.12^1^ |  |
| *Difference (2 vs. 1) p* | <.001 | 0.001 | 0.18^1^ |  |
| **^b^DKA Events** |  |  |  |  |
| No CGM | 0.14 (0.11, 0.16) | 0.32 (0.18, 0.47) | 0.19 (0.04, 0.33) | 0.011 |
| CGM | 0.07 (0.05, 0.08) | 0.09 (0.02, 0.16) | 0.02 (-0.05, 0.09) | 0.51 |
|  |  |  |  |  |
| *Difference p* | <.001 | 0.003 | 0.24^1^ |  |
| No Pump | 0.14 (0.12, 0.16) | 0.33 (0.19, 0.48) | 0.19 (0.05, 0.34) | 0.009 |
| Pump | 0.05 (0.03, 0.07) | 0.08 (0.02, 0.14) | 0.03 (-0.04, 0.09) | 0.42 |
|  |  |  |  |  |
| *Difference p* | <.001 | 0.001 | 0.36^1^ |  |
| No Technology | 0.17 (0.14, 0.20) | 0.40 (0.21, 0.59) | 0.23 (0.04, 0.43) | 0.017 |
| 1 Tech | 0.07 (0.05, 0.09) | 0.13 (0.05, 0.22) | 0.06 (-0.03, 0.15) | 0.17 |
| 2 Tech | 0.05 (0.03, 0.07) | 0.04 (-0.02, 0.09) | -0.01 (-0.07, 0.05) | 0.72 |
|  |  |  |  |  |
| *Difference (1 vs. None) p* | <.001 | 0.012 | 0.57^1^ |  |
| *Difference (2 vs. None) p* | <.001 | <.001 | 0.16^1^ |  |
| *Difference (2 vs. 1) p* | 0.043 | 0.055 | 0.32^1^ |  |
| Note: 1 Tech = Use of CGM or Pump; 2 Tech = Use of both CGM & Pump ^a^Linear regression adjusting for age, sex, and insurance status  ^b^Negative binomial regression adjusting for age, sex, insurance status, and age at first diagnosis (UCLA & UCD) or age at first appearance in EHR(UCSD) ^1^Interaction term testing if CGM/Pump effects are different between depressed and not depressed patients  Est = Marginal estimate from linear regression (HbA1c) or negative binomial regression (DKA events) 95% CI = 95% Confidence Interval  Significant p values are bolded | | | | |
