# Supplementary material for: Correlation Between Technology and Improved Outcomes in Youth With Type 1 Diabetes Mellitus: Prospective Study Examining Outcomes for Patients With Depression and Those With Public Insurance
Source: JMIR Diabetes. 2025 Jun 3;10:e70380. doi: 10.2196/70380 (PMC12151526; doi:10.2196/70380)
Supplement: Multimedia Appendix 2 [file diabetes-v10-e70380-s002.docx]

Table 3. Adjusted effects of Insurance type on HbA1c levels and DKA events by technology use

| **^a^HbA1c** | **Private Insurance** Est (95% CI) | | **Public Insurance** Est (95% CI) | | **Difference** Est (95% CI) | ***p*** |
| --- | --- | --- | --- | --- | --- | --- |
| No CGM | 9.5 (9.4, 9.7) | | 10.0 (9.8, 10.2) | | 0.4 (0.2, 0.7) | **<.001** |
| CGM | 8.5 (8.4, 8.6) | | 8.4 (8.3, 8.6) | | -0.1 (-0.3, 0.1) | 0.35 |
|  |  | |  | |  |  |
| *Difference p* | <.001 | | <.001 | | <.001^1^ |  |
| No Pump | 9.5 (9.3, 9.7) | | 9.7 (9.5, 9.8) | | 0.2 (-0.1, 0.4) | 0.17 |
| Pump | 8.5 (8.3, 8.6) | | 8.7 (8.5, 8.9) | | 0.3 (0.0, 0.5) | **0.026** |
|  |  | |  | |  |  |
| *Difference p* | <.001 | | <.001 | | 0.63^1^ |  |
| No Technology | 9.8 (9.5, 10.0) | | 10.1 (9.9, 10.3) | | 0.3 (0.0, 0.7) | **0.034** |
| 1 Tech | 9.0 (8.8, 9.1) | | 9.0 (8.8, 9.2) | | 0.1 (-0.2, 0.3) | 0.70 |
| 2 Tech | 8.2 (8.1, 8.4) | | 8.2 (8.0, 8.4) | | 0.0 (-0.2, 0.3) | 0.90 |
|  |  | |  | |  |  |
| *Difference (1 vs. None) p* | <.001 | | <.001 | | 0.15^1^ |  |
| *Difference (2 vs. None) p* | <.001 | | <.001 | | 0.10^1^ |  |
| *Difference (2 vs. 1) p* | <.001 | | <.001 | | 0.84^1^ |  |
|  |  | |  | |  |  |
| **^b^DKA Events** | | | | | | |
| No CGM | 0.13 (0.09, 0.17) | | 0.18 (0.14, 0.21) | | 0.05 (-0.00, 0.10) | 0.067 |
| CGM | 0.07 (0.05, 0.09) | | 0.06 (0.04, 0.08) | | -0.00 (-0.03, 0.02) | 0.76 |
|  |  | |  | |  |  |
| *Difference p* | 0.004 | | <.001 | | 0.16^1^ |  |
| No Pump | 0.14 (0.10, 0.17) | | 0.17 (0.14, 0.21) | | 0.04 (-0.01, 0.08) | 0.12 |
| Pump | 0.05 (0.03, 0.07) | | 0.05 (0.03, 0.08) | | 0.00 (-0.03, 0.03) | 0.97 |
|  |  | |  | |  |  |
| *Difference p* | <.001 | | <.001 | | 0.53^1^ |  |
| No Technology | 0.16 (0.11, 0.21) | | 0.21 (0.17, 0.25) | | 0.05 (-0.02, 0.11) | 0.17 |
| 1 Tech | 0.07 (0.05, 0.09) | | 0.09 (0.05, 0.12) | | 0.02 (-0.02, 0.06) | 0.38 |
| 2 Tech | 0.05 (0.03, 0.08) | | 0.04 (0.02, 0.06) | | -0.02 (-0.05, 0.01) | 0.29 |
|  |  | |  | |  |  |
| *Difference (1 vs. None) p* | <.001 | | <.001 | | 0.93^1^ |  |
| *Difference (2 vs. None) p* | <.001 | | <.001 | | 0.11^1^ |  |
| *Difference (2 vs. 1) p* | 0.34 | | 0.009 | | 0.15^1^ |  |
|  |  |  | |  | |  |
| Note: 1 Tech = Use of CGM or Pump; 2 Tech = Use of both CGM & Pump ^a^Linear regression adjusting for age, sex, and insurance status  ^b^Negative binomial regression adjusting for age, sex, insurance status, and age at first diagnosis (UCLA & UCD) or age at first appearance in EHR(UCSD) ^1^Interaction term testing if CGM/Pump effects are different between depressed and not depressed patients  Est = Marginal estimate from linear regression (HbA1c) or negative binomial regression (DKA events) 95% CI = 95% Confidence Interval  Significant *p* values are bolded | | | | | | |
